# Fatal Outcome in a Kidney-Pancreas Transplant Recipient With COVID-19

**DOI:** 10.7759/cureus.8691

**Published:** 2020-06-18

**Authors:** Kulachanya Suwanwongse, Nehad Shabarek

**Affiliations:** 1 Internal Medicine, Lincoln Medical Center, New York City, USA

**Keywords:** novel corona virus, transplant, renal failure, case report, immunocompromised, immunosuppesive, covid-19

## Abstract

Severe acute respiratory syndrome coronavirus 2 (SARS-CoV-2) is a highly contagious pathogen causing the novel coronavirus disease 2019 (COVID-19), the ongoing unprecedented pandemic in 2020. SARS-CoV-2 primarily targets the respiratory systems, so acute respiratory distress syndrome is the major cause of death. Clinical courses of COVID-19 are variable and unpredictable, while some epidemiologic and clinical factors have been found to have a negative impact on the disease prognosis. Despite a growing report on clinical characteristics and prognosis of patients with COVID-19, the data in the special population, including transplant recipients, is still limited. Herein we report on the clinical features and fatal outcome of COVID-19 in a dual pancreas-kidney transplant recipient (with failure of the pancreas graft). Our case illustrates the similarities and differences of the COVID-19 disease course between transplant recipients and the general population. We proposed that the pre-existing T-cell dysfunction from the long-term use of immunosuppressive agents in organ transplant recipients adversely affects COVID-19 prognosis and worsens COVID-19 mortality.

## Introduction

The novel coronavirus disease 2019 (COVID-19), caused by severe acute respiratory syndrome coronavirus 2 (SARS-CoV-2), is one of the greatest pandemic challenges in the 21st century. Since its first report in December 2019, SARS-CoV-2 has rapidly spread worldwide resulting in over five million confirmed cases and almost 400,000 deaths [1].

In general, transplant recipients are vulnerable to develop serious infections due to chronic medical-induced immunosuppressive states. However, impaired immune functions may paradoxically protect transplant patients from the hyper-inflammatory response to SARS-CoV-2 and thus dampen the disease severity. Previous reports on the outcomes of COVID-19 in transplant recipients showed contradictory results, in which the mortality rate ranges from 0% to 67% [2-9]. The diversity in COVID-19 prognoses across each literature may be due to different baseline patient characteristics, including age, sex, and comorbidities, as well as various management strategies across institutions. Herein we report our experience in treating COVID-19 in a dual pancreas-kidney transplant recipient and discuss the current knowledge regarding COVID-19 in transplant patients.

## Case presentation

We present the case of a 52-year-old male who received dual pancreas-kidney transplantation from a deceased donor 20 years ago because of end-stage renal disease (ESRD) due to diabetic nephropathy. He had pancreas graft failure and underwent second pancreas transplantation 12 years ago with immediate graft failure from thrombosis and third pancreas transplantation 11 years ago with failure of the graft a year later due to viral infection. He then decided not to have further attempts of pancreas transplantation.

His maintenance immunosuppressive therapy included oral tacrolimus (TAC) 4 mg twice daily, mycophenolate mofetil (MMF) 250 mg twice daily, and prednisone 5 mg once daily. He reported medication compliance and was adherent to follow-up appointments. His baseline creatinine ranged 2.0-2.5 mg/dl over the past year. The decline in his renal function was due to diabetic nephropathy, which was diagnosed by renal biopsy. His most recent serum creatinine (Cr), two months prior to the current admission, was 2.34 mg/dl. The patient also had insulin-dependent diabetes mellitus (recent hemoglobin A1C 7.8%), hypertension, and obesity (body mass index 30.18 kg/m^2^), for which he took losartan 50 mg once daily, metoprolol succinate 25 mg once daily, rosuvastatin 5 mg once daily, and insulin regimens.

**Figure 1:**
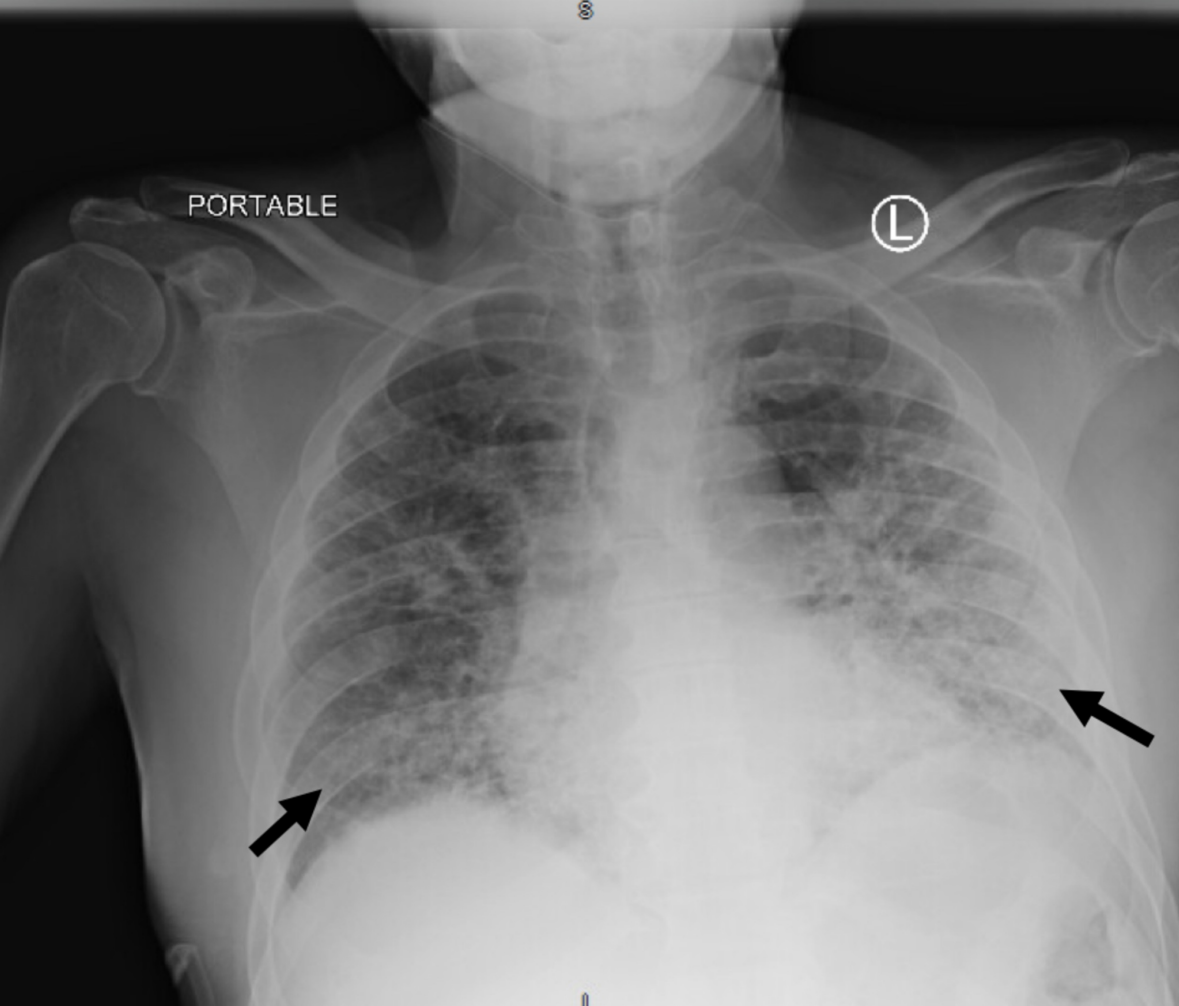
Chest x-ray showing bilateral multifocal ground-glass appearances

He presented with worsening dyspnea for three days and reported subjective fever, dry cough, watery diarrhea, polydipsia, and polyuria. On initial evaluation, his temperature was 97.8 °F, blood pressure was 145/78 mmHg, heart rate was 112 beats per minute, and respiratory rate was 28 breaths per minute with oxygen saturation (SpO_2_) of 92% on room air. He had dry mucous membranes and did not appear to use accessory respiratory muscles. His lung examination revealed rales in both lungs. His neurological examination was normal. Chest x-ray (CXR) showed bilateral multifocal ground-glass appearances (Figure 1). Laboratory values are described in Table 1.

**Table 1:** Initial laboratory results of the patient

Laboratory results	
Leukocytosis	White blood cell count 12,200/mcl
Lymphopenia	Lymphocytes 5.9%
Neutrophilia	Neutrophils 85.7%
Creatinine	1.54 mg/dl
Hypokalemia	Potassium 2.7 mmol/l
Acidemia	Venous blood gas pH 7.26, pCO_2_ 41.7 mmHg
Hepatic function panel	Normal
Inflammatory markers significantly elevated	C-reactive protein 9.22 mg/l, D-dimer 996 ng/ml, ferritin 569 ng/ml
Ketonemia (beta-hydroxybutyrate >4.40 mmol/l, normal <0.27 mmol/l) and ketonuria	

SARS-CoV-2 reverse transcriptase-polymerase chain reaction (RT-PCR) from the nasopharyngeal swab specimen was positive. He was diagnosed with diabetic ketoacidosis (DKA) and COVID-19. Due to a lack of a negative-pressure room in our institution, he was admitted to a regular patient room on the medicine floor, with droplet and contact precaution. The patient received the standard treatment of DKA, including insulin drip, aggressive electrolytes replacement, and intravenous hydration. He received supplemental oxygen via nasal cannula (4 l/min), and his SpO_2_ was improved to 99%-100%. Hydroxychloroquine (400 mg twice daily on the first day, and 200 mg twice daily for the following four days) was administered for COVID-19 treatment as per hospital protocol. Assuming his immunocompromised status, the primary team decided to give antibiotics: oral doxycycline and intravenous ceftriaxone for possible secondary bacterial pneumonia. His TAC was stopped. MMF and prednisone were continued with the same dosage. His blood pressure medications and statin were discontinued.

The next day, his DKA was resolving. However, he developed acute hypoxemic respiratory failure requiring invasive ventilation. His arterial oxygen partial pressure (PaO_2_) to fractional inspired oxygen (FiO_2_) (P:F) ratio was 69 indicating severe acute respiratory distress syndrome (ARDS). Blood tests showed serum creatinine (Cr) rising to 2.49 mg/dl. He was transferred to our COVID-19 ventilator unit for intensive care. On Day 3, he developed septic shock requiring small doses of intravenous norepinephrine drip (range 2-10 mcg/min). His prednisone was discontinued, and he was started on stress dose corticosteroids: intravenous hydrocortisone 50 mg every six hours.

On Day 5, his Cr worsened to 3.38 mg/dl, while his respiratory function remained stable. The primary team contacted his transplant center for possible transfer due to suspected acute graft rejection from discontinuation of TAC. However, he was rejected due to a lack of patient beds. TAC was re-started. The next day, the patient became more hypoxemic with a P:F ratio of 54. His SpO_2_ was around 80% despite adjusted high ventilator setting: volume control - positive end-expiratory pressure (PEEP) value of 20 mmHg and 100% FiO_2_. CXR showed worsening pneumonia and ARDS. He was started on the proning protocol, with mild improvement of SpO_2^ ^_to 85%-88%. Although his hypoxemia was likely from severe ARDS, the primary team decided to give enoxaparin to treat possible pulmonary embolism, assuming the medication risks were lower than benefits.

**Figure 2:**
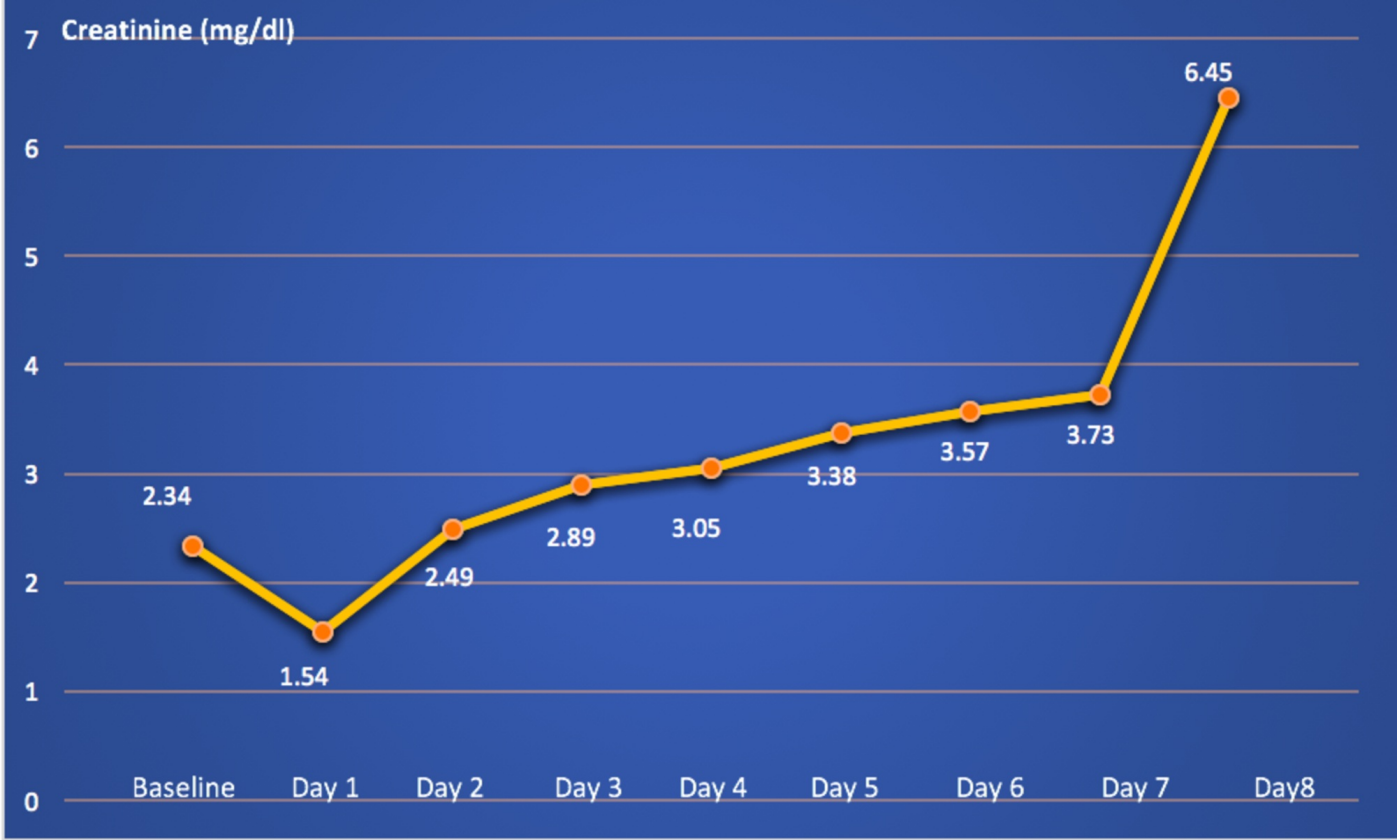
Changes in patient's creatinine level during hospitalization

On admission day 8, he acutely developed oliguria with urine output less than 50 ml in eight hours. His creatinine was doubled to 6.45 mg/dl. His transplant center was contacted again, but there was still no bed available. The daily changes in his creatinine are demonstrated in Figure 2. He received hemodialysis with total ultrafiltration of 2 l. However, his conditions were progressively worsening and he eventually died on Day 11. Multiple blood and sputum cultures were done during hospital courses, which all revealed negative results. His cause of death was septic shock and multi-organ failure from severe COVID-19.

## Discussion

SARS-CoV-2 uses angiotensin-converting enzyme 2 (ACE2) receptors to enter the host cells causing a spectrum of diseases ranging from flu-like illness, pneumonia to severe ARDS, septic shock, and death [10,11]. The uncontrolled hyper-inflammatory response to SARS-CoV-2, rather than the own viral virulence, presumably underlies the COVID-19 severity [12,13]. Certain comorbidities, including hypertension, diabetes mellitus, and cardiovascular diseases, adversely impact COVID-19 prognosis, probably due to the changes in the renin-angiotensin system (RAS) or underlying chronic inflammatory states [11,14]. Many research teams have been repurposing various anti-viral and immunomodulating agents to treat COVID-19. However, to date, no specific drugs have proven mortality benefits, and unfortunately, the best COVID-19 management is supportive measures, which include the provision of supplemental oxygen, invasive ventilation, and extracorporeal membrane oxygenation [15].

Long-term immunosuppressive therapy in organ transplant recipients may alter clinical features and outcomes of COVID-19. However, considering our patient and previously published literature, transplant recipients with COVID-19 did not show prominent differences in terms of clinical symptoms, laboratory values, and CXR findings [2-9].

Our patient had hypertension, diabetes mellitus, and obesity, which are identified as poor prognostic markers of COVID-19 in the normal population [11,14]. Whether these comorbidities negatively impact COVID-19 in transplant recipients is not well-characterized, but most of the literature published so far and our case report suggest a similar result [2-9]. Recent evidence showed that the duration of organ transplantation correlates with COVID-19 severity [16]. Our patient underwent dual kidney-pancreas transplantation for 20 years, so he had unfavorable outcomes leading ultimately to death. Whether the type and number of organ transplantation affect the COVID-19 courses remains undetermined.

The long-term use of immunosuppressive medications in organ transplant recipients is associated with the decrease in T-cell number and function; TAC and MMF preferentially inhibit T-cell response. T-cell lymphocytopenia has also been found in patients with COVID-19 and is correlated with the disease severity [17]. Whether the chronic T-cell suppression in organ transplant recipients attenuates the clinical course and severity of COVID-19 remains unknown. Recently, we reported the extremely high mortality rate of HIV patients with SARS-CoV-2 infection [18]. T-cell impairment is the major characteristic of HIV-associated immunopathology. Based on our observations, we propose that the previous medically induced T-cell impairment negatively affects COVID-19 prognoses in the transplant recipients. Further research exploring the role of T-cell lymphocytes in SARS-CoV-2 pathogenesis will be worthwhile.

It is uncertain whether the immunosuppressive agents should be adjusted in transplant recipients who have been infected with SARS-CoV-2. Aberrant host-immune reactions characterized by hyper-inflammatory states and persistent cytokines releases likely contributed to COVID-19 severity. Severe COVID-19 patients had considerably high serum pro-inflammatory cytokines, including tumor necrosis factor (TNF)-α, interleukin (IL)-1, and IL-6 [13]. Abrupt discontinuation of immunosuppressive agents may lead to an exaggerated rebound inflammatory response to SARS-CoV-2. A study from the Netherlands found that immunosuppressive drug withdrawal worsened COVID-19 mortality. However, in this report, immunosuppressive agents were discontinued in patients with severe disease, presumably with high mortality risks. The current expert opinion urges adjusting the immunosuppressive agents based on COVID-19 severity. We have summarized the recommendations in Table 2 [19].

**Table 2:** Recommendations regarding the adjustment of immunosuppressive regimens in transplant recipients with COVID-19 COVID-19, coronavirus disease 2019; MPA, mycophenolic acid; AZA, azathioprine; mTOR, mammalian target of rapamycin; CNI, calcineurin inhibitor. CNIs include tacrolimus and cyclosporine.

Severity/regimen	Triple therapy	Dual therapy (steroids)	Dual therapy (no steroids)
Mild	Stop MPA/AZA/mTOR	Continue same regimens	Consider replacing MPA/mTOR with low-dose steroids
Moderate	Stop MPA/AZA/mTOR; consider stopping or decreasing CNIs; consider maintenance steroids or increase to 15-25 mg/day		
Severe	Discontinue all immunosuppressive drugs; steroids 15-25 mg/day		

Surprisingly, on admission, our patient had an increase in the glomerular infiltration rate. This is likely due to the early phase of sepsis and acute kidney injury (AKI). Clinicians should be aware that patients may have lower serum Cr during the early phase of AKI. Other common causes of the acute drop in Cr include urinary tract obstruction and certain medication effects. The doubled rise of his Cr level on Day 8 was likely due to septic shock and multi-organ failure. The patient was unlikely to have acute renal graft rejection from TAC withdrawal as he had undergone transplantation for more than 10 years and TAC was discontinued for only a few days. The definitive diagnosis of acute renal graft rejection requires kidney biopsy. However, it was not done in our patient as the diagnosis would not change our management. The patient required hemodialysis as he had oliguric acute renal failure and suffered from severe COVID-19.

## Conclusions

We report a clinical course and lethal outcome of COVID-19 in a transplant recipient. Pre-existing T-cell immune response deficits from long-term use of immunosuppressive agents may worsen the prognosis of COVID-19 in transplant recipients. The optimal management of COVID-19 in the post-transplant population remains unclear. The recent expert opinion recommended adjusting immunosuppressive agents based on COVID-19 severity. There is an urgent need to develop the collaborative management guidelines of organ transplant recipients with COVID-19 in the non-transplant centers to provide patients the best available care.
